# Insulin and risk of diabetic retinopathy in patients with type 2 diabetes mellitus: data from a meta-analysis of seven cohort studies

**DOI:** 10.1186/1746-1596-9-130

**Published:** 2014-06-27

**Authors:** Chun Zhao, Weifang Wang, Ding Xu, Hui Li, Min Li, Fang Wang

**Affiliations:** 1 Department of Ophthalmology, Affiliated Tenth People’s Hospital of Tongji University, Shanghai 200072, China; 2 Affiliated Shanghai Tenth Clinical Medical College of Nanjing Medical University, Nanjing, Jiangsu, China

**Keywords:** Type 2 diabetes mellitus (T2DM), Insulin, Diabetic retinopathy (DR), Meta-analysis

## Abstract

**Background:**

Type 2 diabetes mellitus (T2DM) is a chronic incurable disease associated with multi-systemic complications. The chronic complications related to T2DM induce growing burden to the national health system. Diabetic retinopathy (DR) is the most serious ocular complication associated with T2DM and one of the leading causes of secondary blindness. The association between insulin use and DR risk has also been reported in different studies.

**Methods:**

In order to obtain more informative results on the relationship between insulin intake and risk of DR and to take into account more recent evidence, we conducted this meta-analysis by including all available relevant cohort studies. A systemic literature search was performed via electronic databases inclu-apding Pubmed and EMBASE to identify all available relevant studies until February 2014. A total of seven cohort studies were included in this meta-analysis. In this meta-analysis, we conducted a rigorous search of all available published cohort studies to quantify the possible association between insulin use and incidental DR in individuals with type 2 diabetes.

**Results:**

Although major heterogeneity existed in this study, the significant association between insulin use and risk of DR was detected. The subgroup analyses by study design, region, data source and adjustment of HbA1c generated similar results. Also, when the DM duration was adjusted, no result was reported with significant difference.

**Conclusion:**

The results of this meta-analysis helps to better explore the role of insulin use in DR risk development. Meanwhile, our results are statistically robust and yield important conclusions. The underlying mechanism by which insulin use increases DR risk should be explored in future in vitro and in vivo studies. Additional large-scale, well-designed studies with sufficient data are needed to confirm our findings.

**Virtual Slides:**

The virtual slide(s) for this article can be found here: http://www.diagnosticpathology.diagnomx.eu/vs/2003724731291657

## Background

Type 2 diabetes mellitus (T2DM) is a chronic incurable disease associated with multi-systemic complications. It is also a significant and growing source of morbidity and mortality in the whole world [1,2]. The prevalence of T2DM has rapidly increased in the past decades worldwide, particularly in developing countries where people are most vulnerable in confronting this complex and serious disease. Nowadays, increasing concern has been attracted on T2DM because of the serious consequences caused by both the disease itself and its complications. For instance, chronic complications related to T2DM not only induce growing burden to the national health system and increasing rate of diabetes-related disability, but also result in untimely mortality as well as lowered life quality. Diabetic retinopathy is the most serious ocular complication associated with T2DM and one of the leading causes of secondary blindness [3]. The prevalence of DR is reported to be ranging from 15.3% to 42.4% in different epidemiologic studies. Different risk factors of DR have been investigated and reported. Previous studies reported that both modifiable risk factors (blood glucose, blood pressure, serum lipids, and smoking) and non-modifiable risk factors (duration, age, genetic predisposition, and ethnicity) are responsible for DR progression [4-6].

Insulin is one of the most important therapeutic measures in the treatment of DM. Recently, the use of insulin has been reported to be associated with various diseases, such as hypertension, limb ischemia and diverse types of cancers [5-8]. For example, a prospective study was conducted to investigate the association between insulin use and colorectal cancer (CRC) risk [7]. The association between insulin use and DR risk has also been reported in different studies. In a cross-sectional, multi-centered, hospital-based study, the results showed that there was more prevalent non-proliferative diabetic retinopathy (NPDR) and proliferative diabetic retinopathy (PDR) in insulin-taking than those in non-insulin-taking groups [8]. Admittedly, each cross-sectional study design would induce certain bias and limitations, thus weakening its reliability. In order to obtain more informative and reliable results on the relationship between insulin intake and risk of DR and to take into account more recent evidence, we conducted this meta-analysis by including all the available relevant cohort studies [9].

## Methods

### Search strategy

We conducted the present meta-analysis following the Preferred Reporting Items for Systematic Reviews and Meta-Analyses (PRISMA) statement [10] and meta-analysis of observation studies in epidemiology (MOOSE) guidelines [11]. A systemic literature search was performed via electronic databases including Pubmed and EMBASE to identify all available relevant studies until February 2014. Manual retrieval of reference lists from retrieved articles and reviews was also conducted. Medical subject heading terms and key words used in the search included “hypoglycemic agents”, “insulin” combined with “diabetic retinopathy”. No language or other restrictions were set in this study.

### Study selection

Two authors (CZ and WFW) independently reviewed the title and abstract of each study identified in the primary searching process and excluded the studies that did not answer the research question of interest. The full texts of the remaining articles, including the references, were carefully examined to determine whether relevant information did exist inside. Disagreement between the two reviewers was settled by discussing with the third reviewer (FW). Studies were selected if they met the following criteria: (1) a cohort study design was obtained; (2) the association between insulin use and DR risk was reported; (3) Studies reporting different measures of RR like risk ratio, rate ratio, hazard ratio (HR), and odds ratio (OR) were reported.

### Data extraction

The data extraction was conducted by two researchers (CZ and WFW) independently and the following data was extracted from each included study: the first author’s last name, year of publication, geographic location(s), number of all the enrolled subjects and cases, study design, data source, duration of follow-up in cohort studies, confounders for adjustment, and effect size estimates with corresponding 95% CIs of all the enrolled papers. In studies where more than one estimate of effect was reported, we chose the ‘most adjusted’ estimate in this study.

### Methodological quality assessment

We used Newcastle-Ottawa Scale (NOS) to assess the methodological quality of the enrolled cohort studies. The NOS contains eight items that are classified into three categories: selection (four items, one star each), comparability (one item, up to two stars), and outcome (three items, one star each). A “star” presents a “high-quality” choice of individual study. Two reviewers (CZ and WFW) assessed the methodological quality independently. Disagreement between the two reviewers was settled by discussing with the third reviewer (FW).

### Statistical methods

We used the method of a random-effect model to calculate summary RR and 95% CIs for assessing the association between insulin use and risk of DR. The square of the SEM was used as the estimated variance of the logarithm of the OR. Only a random-effect model was assessed, regardless of the significance of the heterogeneity. Heterogeneity was assessed using the χ^2^ and I^2^ statistics. For the χ^2^ statistic, a P value < 0.10 was considered statistically significant for heterogeneity; for the I^2^ statistic, heterogeneity was interpreted as absent (I^2^: 0%–25%), low (I^2^: 25.1%–50%), moderate (I^2^: 50.1%–75%), or high (I^2^: 75.1%–100%), respectively. The subgroup analyses were performed according to the following indexes including study design (prospective cohort or retrospective cohort), data source (population based or hospital based), study population (Europe, America and Asian), and control for confounding factors. All statistical analyses were performed using STATA, version 12.0 (STATA, College Station, TX). A two-tailed p value < 0.05 was considered to be statistically significant.

## Results

### Literature search results

**Figure 1:**
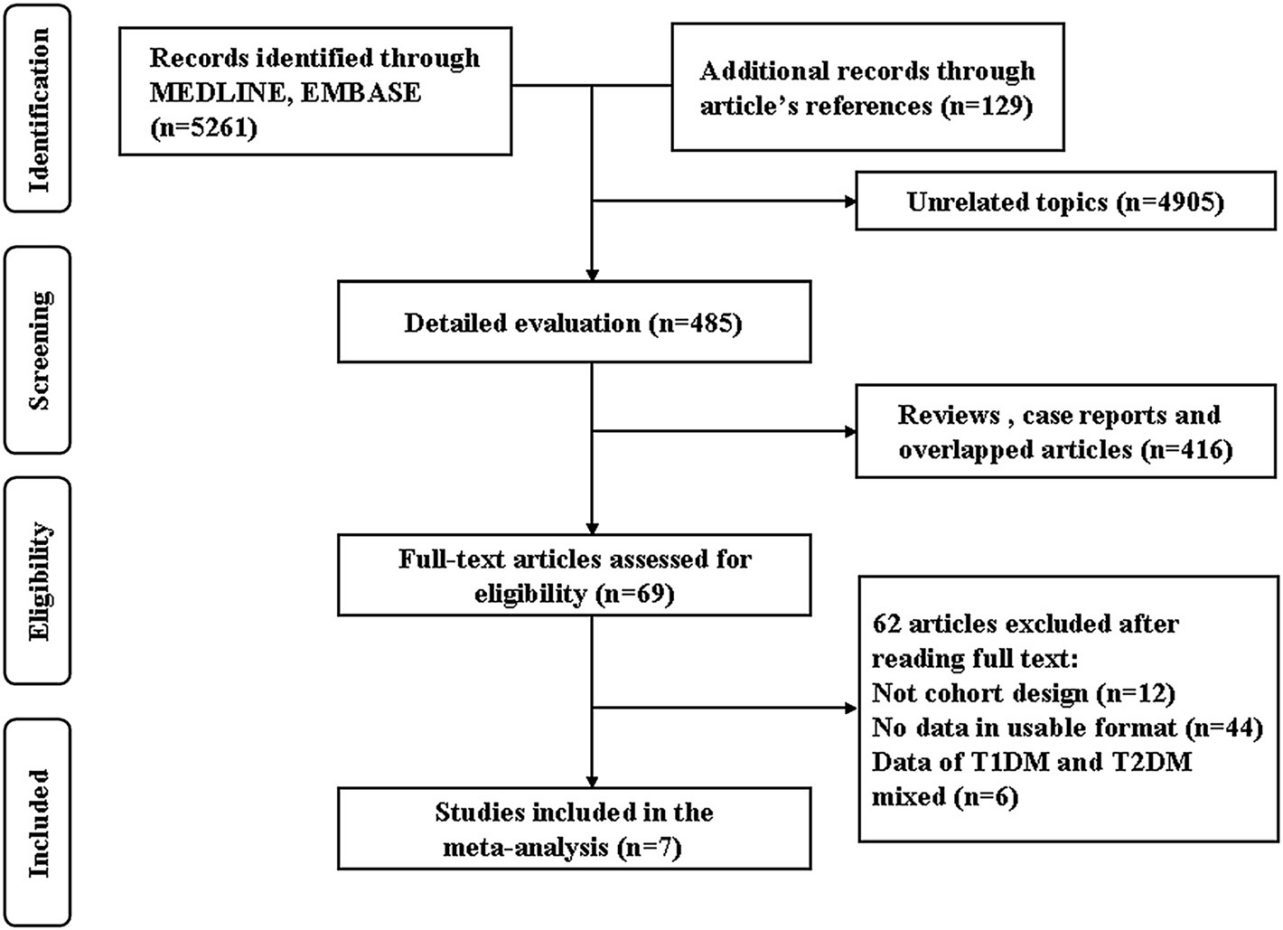
Flow diagram of screened, excluded, and analyzed publications.

A total of 5261 unique studies were yielded using the previously mentioned search strategy in Medline and EMBASE electronic databases until February 2014 and 129 additional studies were identified from the references of the retrieved articles (Figure 1). In all, 4905 articles were found not to be involved in the association between insulin intake and DR risk. From the 485 studies that have been evaluated carefully, 416 reviews, case reports and overlapped articles were further excluded. Out of 69 articles that examined the association between insulin use and DR risk, 12 studies were excluded because of not being cohort design-based studies, 44 studies were excluded because of no data available in usable format, and 6 studies were excluded because of the mixture of T1DM and T2DM. As a result, a total of seven cohort studies were finally included in this meta-analysis [12-18].

### Characteristics of studies included in the meta-analysis

The characteristics of these studies included in this meta-analysis are shown in Table 1. Among all the studies, a total of 1711 cases including 19107 subjects were identified. The earliest study was launched in 1967, and the latest ended in 2010. Six of these studies were population-based studies, and the remainder one study was a hospital-based study. Among all the studies, 5 studies were conducted in Europe, 1 in America and 1 in Asia. The records of all the studies included both male and female cases. The confounders for adjustments of each study were presented in Table 1.

**Table 1:** Characteristics of the study population included in this study

**Study**	**Year of publication**	**Study design**	**Data source**	**Country**	**All subjects**	**DR cases**	**Study period**	**Sex**	**Confounders for adjustment**
Gunnlaugsdottir E	2012	Prospective	Population based	Iceland	4,995	138	1967-1997	M/F	Age, sex, systolic BP. duration of DM, oral hypoglycaemic, HbA1c, hypertension and microlbuminuria
Geir Bertelsen	2012	Prospective	Population based	Norway	514	110	2007-2008	M/F	Age, sex, systolic BP, oral hypoglycaemic, HbA1c, hypertension and microlbuminuria, BMI, glaucose
Schweitzer K	2009	Prospective	Population based	American	500	175	2004-2007	M/F	NA
Romero-Aroca P	2007	Prospective	Hospital based	Spain	741	205	2005.1-2005.12	M/F	NA
Hove MN	2004	Restropective	Population based	Denmark	10,851	378	2000.1-2000.12	M/F	NA
Henricsson M	1996	Prospective	Hospital based	Sweden	1,378	438	1990-1995	M/F	Age, sex and duration of diabetes
Deng Y	2014	Prospective	Population based	China	128	267	2009-2010	M/F	Age of diabetic onset, duration of diabetes, BMI, microalbuminuria,HbA1c, fasting plasma glucose, creatinine

NA, not applicable; M: male; F: female; BP: blood pressure; BMI: body bass index; DR: diabetic retinopathy.

### Quality of included studies

To evaluate the methodological qualities of the included studies, the Newcastle-Ottawa quality tool was used in the current meta-analysis. The Newcastle-Ottawa quality assessment score of the most studies (mean: 6.86; standard deviation: 1.21) and all the studies were at a relatively high level, indicating a high methodological quality of the enrolled studies (Table 2).

**Table 2:** **Quality assessment of included studies**^**1**^

**Author**		**Quality assessment criteria**			
		**Selection**	**Comparability**	**Outcome**	**Overall quality**
Gunnlaugsdottir E	2012	**	**	**	6
Geir Bertelsen	2012	**	**	**	6
Schweitzer K	2009	***	**	***	8
Romero-Aroca P	2007	**	**	***	7
Hove MN	2004	***	**	**	7
Henricsson M	1996	**	**	**	6
Deng Y	2014	***	**	***	8

^1^the Newcastle-Ottawa Scale (NOS) was obtained to assess the methodological quality of the included studies.

**Two points; ***Three points.

### Quantitative synthesis

**Figure 2:**
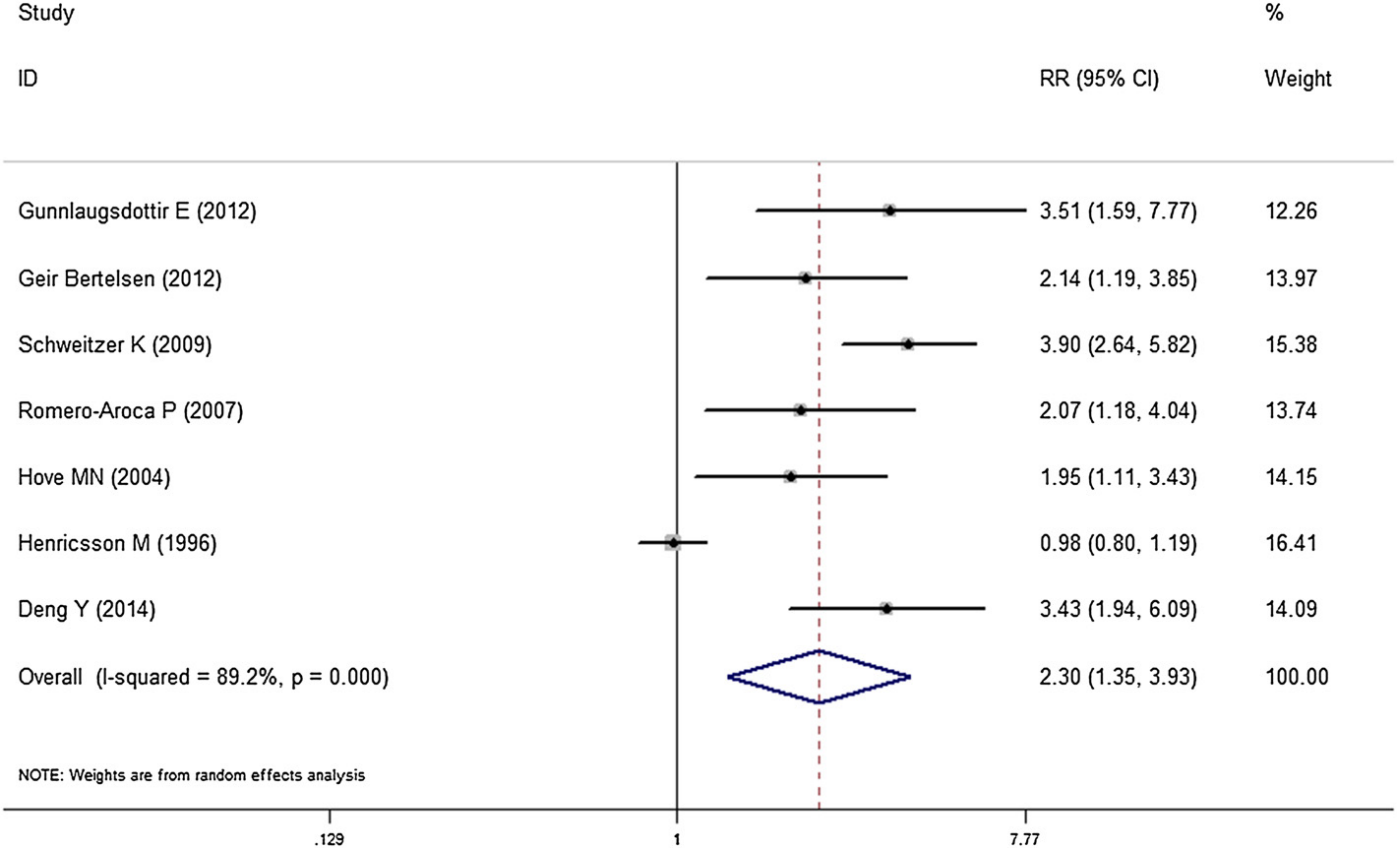
**Forest plot: overall meta-analysis of insulin use and DR risk.** Squares indicated study-specific risk estimates (size of square reflects the study-statistical weight, i.e. inverse of variance); horizontal lines indicate 95% confidence intervals; diamond indicates summary relative risk estimate with its corresponding 95% confidence interval.

Figure 2 displayed the pooled associations between insulin use and DR risk. The pooled result of all the 7 included studies showed that insulin use is associated with increased risk of DR (RR = 2.30, 95% CI, 1.35-3.93). Table 3 displayed the effects of insulin use and DR risk in subgroup analysis by adjusting confounding factors including status, study type, country, and study designs. In both prospective studies (RR, 2.38; 95% CI, 1.28-4.41) and retrospective studies (RR, 2.30; 95% CI, 1.11-3.43) subgroups, a significant correlation between insulin use and incidence rate of DR was observed. When subgroup analyses were conducted according to the geographic locations, significant associations were detected in Europe (RR, 1.85; 95% CI, 1.12-3.08), Asia (RR, 3.43; 95% CI, 1.93-6.08) and America (RR, 4.90; 95% CI, 2.63-5.79). Moreover, similar results were yielded in the subgroup analyses by data source (population based or hospital based), follow-up duration (over 5 years or less than 5 years) and adjustments of diabetic status (HbA1c adjusted or HbA1c not adjusted). When DM duration was adjusted, no significant association between insulin use and risk of DR was detected (RR, 2.18; 95% CI, 0.80-5.93).

**Table 3:** Detailed outcomes on insulin use and RRs of DR

**Subgroups**	**No. of studies**	**Pooled estimate**		**Tests of heterogeneity**	
		**RR**	**95% CI**	**P value**	**I**^**2**^**(%)**
All subjects	7	2.30	1.35-3.93	< 0.001	89.3
Study design					
Prospective study	6	2.38	1.28-4.41	< 0.001	90.9
Retrospective study	1	2.30	1.11-3.43	—	—
Geographic location					
Europe	5	1.85	1.12-3.08	< 0.001	80.4
Asia	1	3.43	1.93-6.08	—	—
North America	1	4.90	2.63-5.79	—	—
Data source					
Population based	5	2.94	2.19-3.94	0.234	28.1
Hospital based	2	1.34	0.65-2.76	0.024	80.5
Follow-up duration					
≥ 5 years	1	3.51	1.59-7.76	—	—
< 5 years	6	2.17	1.22-3.85	< 0.001	90.3
Major confounders adjusted					
DM duration					
Yes	3	2.18	0.80-5.93	< 0.001	91.7
No	4	2.53	1.74-3.67	0.115	49.4
HbA1c					
Yes	3	2.88	2.00-4.14	0.454	0
No	4	2.30	1.35-4.14	< 0.001	92.7

RR: relative risk; CI: confidence interval; DM: diabetes mellitus.

### Heterogeneity and publication bias

A significant heterogeneity was observed when all the 7 cohort studies were included (*I*^*2*^, 89.3%; *P* < 0.001). In the subgroup analyses, the heterogeneity remains significant. However, when one study by Henricsson M et al. [16] was excluded from the meta-analysis, (*I*^*2*^, 26.3%; *P* = 0.237), only low heterogeneity was observed. The differences in the data source or regional characteristics might be responsible for generating a significant heterogeneity. However, the heterogeneity remains significant when that study was excluded (RR, 2.86; 95% CI, 2.28-3.58).

No significant publication bias was found in the 7 enrolled studies (Begg’s test, *P* for bias = 0.30; Eegg’s test, *P* for bias = 0.297).

## Discussion

In this meta-analysis, we conducted a rigorous search of all available published cohort studies to quantify the possible association between insulin use and incidental DR in individuals with type 2 diabetes. Although a dramatic heterogeneity existed in this study, the significant association between insulin use and DR was detected. The subgroup analyses by study design, region, data source and adjustment of HbA1c yielded similar results. In the group when the DM duration was adjusted, no significant result was reported.

Insulin and DR risk have been discussed for long. Several cross-sectional studies have reported that insulin use is a risk factor for DR. In certain cross-sectional studies, the association between insulin use and risk of DR was reported [15,16]. According to data from the Tromsø Eye Study, a study including 514 participants with diabetes aged from 46 to 87 years, showed that DR risk was associated with insulin use (OR 2.14, 95% CI 1.19-3.85) [17]. In another cross-sectional study performed among 261 type 2 diabetic patients at Chandrubeksa Hospital on January 2011, the result demonstrated that the patients who had received insulin treatment were more likely to suffer from DR than those who had not (OR 3.95, 95% CI 1.86, 8.39) [19]. However, considering that various potential sources would be involved in cross-sectional study design, a cohort study design would be preferred for yielding less potential bias. Meta-analysis is now a useful statistical tool to pool relevant studies together and gain a more powerful conclusion. The meta-analysis was also used in the search for potential risk factors for DR. Zhang et al. reported that in a meta-analysis including nine studies with 1, 217 cases and 1, 459 controls, 4G/5G polymorphism in the PAI-1 gene potentially increased the risk of DR in type 2 diabetes and showed a discrepancy between different ethnicities [20]. In this study, we conducted a meta-analysis only including cohort studies to investigate the association between insulin use and risk of DR. The high methodological quality of included studies and powerful statistical tool employed in this meta-analysis combinely supported the reliability of the presented robust conclusion.

In the subgroup analyses, we found that the association between insulin use and DR risk became non-significant when the DM duration was adjusted. It suggested that the increasing risk of DR in insulin users might be associated with a longer DM duration, while DM duration was generally accepted as a risk factor for DM [19,20]. In the subgroup analyses by other confounding factors, no different results were found. The underlying mechanism of the association between insulin use and risk of DR should be further explored in more studies. In our meta-analysis with seven cohort studies included, we pointed that insulin use might be a risk factor of DR. This finding pointed that we should be more discreet in the patients with long time insulin use history and the DR detection should be more careful. Besides, as insulin use is reported to be risk factor of different diseases [21,22], any inapposite insulin use in the treatment of patients should be avoided.

Only cohort studies were enrolled in the present meta-analysis, which helps to add strength to our study. Besides, access to adequate literature and detailed analyses of the outcome also provide us with detailed understanding of the correlation between insulin use and DR risk. However, limitations of this meta-analysis should also be noted. First, among all the included studies, not enough studies provided the data of insulin use and NPDR or PDR risk, which make it difficult to further explore the role of insulin take in DR onset and progression. Second, considering that only seven cohort studies were included, the power of the conclusion was relatively limited. The third limitation is that most studies lack a long enough follow-up duration and thus more studies are needed to confirm our conclusion.

## Conclusion

Despite these aforementioned limitations, the results of this meta-analysis provide a more complete and systematic picture of the role of insulin use in the development DR risk. Meanwhile, our results are statistically robust and yield important conclusions. The mechanisms by which insulin use increases the DR risk should be further explored in future in vitro and in vivo studies. Additional large-scale, well-designed studies with sufficient data are still wanted to confirm our conclusion.

## Competing interests

The authors declare that they have no competing interests.

## Authors’ contributions

CZ, WFW and FW conceived the study idea and designed the study. CZ, WFW, DX, HL, ML and FW reviewed the literature and performed statistical analyses. CZ, WFW and DX extracted data and drafted the manuscript. CZ, WFW, DX, HL, ML and FW reviewed and edited the manuscript. All authors read and approved the final manuscript.

## Authors’ informations

Chun Zhao and Weifang Wang are co-first author.
